# Carbon Molecular Sieve Membranes Comprising Graphene Oxides and Porous Carbon for CO_2_/N_2_ Separation

**DOI:** 10.3390/membranes11040284

**Published:** 2021-04-12

**Authors:** Chong Yang Chuah, Junghyun Lee, Juha Song, Tae-Hyun Bae

**Affiliations:** 1 Singapore Membrane Technology Centre, Nanyang Environment and Water Research Institute, Nanyang Technological University, Singapore 637141, Singapore; chongyang.chuah@ntu.edu.sg; 2 School of Chemical and Biomedical Engineering, Nanyang Technological University, Singapore 637459, Singapore; junghyun002@e.ntu.edu.sg (J.L.); songjuha@ntu.edu.sg (J.S.); 3 Department of Chemical and Biomolecular Engineering, Korea Advanced Institute of Science and Technology, Daejeon 34141, Korea

**Keywords:** graphene oxide, activated carbon, carbon molecular sieve membrane, carbon capture, gas permeation test

## Abstract

To improve the CO_2_/N_2_ separation performance, mixed-matrix carbon molecular sieve membranes (mixed-matrix CMSMs) were fabricated and tested. Two carbon-based fillers, graphene oxide (GO) and activated carbon (YP-50F), were separately incorporated into two polymer precursors (Matrimid^®^ 5218 and ODPA-TMPDA), and the resulting CMSMs demonstrated improved CO_2_ permeability. The improvement afforded by YP-50F was more substantial due to its higher accessible surface area. Based on the gas permeation data and the Robeson plot for CO_2_/N_2_ separation, the performances of the CMSMs containing 15 wt % YP-50F and 15 wt % GO in the mixed polymer matrix surpassed the 2008 Robeson upper bound of polymeric membranes. Hence, this study demonstrates the feasibility of such membranes in improving the CO_2_/N_2_ separation performance through the appropriate choice of carbon-based filler materials in polymer matrices.

## 1. Introduction

Despite efforts to mitigate the dependence on non-renewables through the creation of alternative energy sources, technical challenges such as intermittency and high cost of energy production have hampered their practical usefulness thus far [1,2]. Meanwhile, considering the fact that the atmospheric CO_2_ concentration had surpassed 400 ppm in 2013 [3,4,5], the release of this potent greenhouse gas into the atmosphere during the combustion process should be minimized. To this end, carbon capture and sequestration (CCS) has been proposed. In post-combustion CO_2_ capture, typically 70% of the total cost of CCS operation is derived from the CO_2_ capture step [6,7,8,9]. Therefore, more effective protocols are necessary to capture CO_2_ at the lowest possible cost. In industrial processes, amine scrubbing, which depends on nucleophilic substitution reaction between amine and CO_2_, is considered as one of the most reliable CCS technologies [10,11]. However, such process remains limited by the high energy penalty. This is associated with the high isosteric heat of adsorption (−50 to −100 kJ/mol) at low CO_2_ loading and the large volume of water in the sorbent [12]. As an alternative, the use of membranes for gas separation is considered a highly feasible approach due to the small plant footprint and high energy efficiency. In principle, polymeric membranes are ideally suited to this technology due to a well-established synthesis procedure and the feasibility of developing membranes in various configurations. Nevertheless, a trade-off relationship between permeability and selectivity has been reported, as expressed by the Robeson upper bound [13,14], arising from the fact that gas transport through polymeric membrane occurs via solution-diffusion process.

Meanwhile, the carbon molecular sieve membrane (CMSM), which is fabricated via carbonization of polymeric precursors, has demonstrated the potential to improve gas separation performances. CMSMs can be prepared by a variety of treatment process (e.g., under an inert gas or in a vacuum) [15,16,17]. However, their performance is highly dependent on the pyrolysis operating parameters during fabrication and the initial choice of polymer precursors [18,19]. Besides, it is worth noting that synthesis of 6FDA-based polyimides and PIM-1, which possess high intrinsic gas permeabilities, typically requires extensive monomer purifications to increase the molecular weight [20]. This hampers the potential large-scale production of these polymers. Meanwhile, the gas separation performance of CMSMs could also be enhanced by the appropriate choice of mixed-matrix membranes with the addition of suitable porous fillers. The mixed-matrix CMSMs fabricated by such a process could be expected to show substantial performance enhancements, with the possibility of overcoming the trade-off relation between permeability and selectivity.

In this study, mixed-matrix CMSMs were fabricated via the incorporation of two different types of carbon-based fillers, which are graphene oxide (GO) and activated carbon (YP-50F) into the polymeric membranes. GO is typically developed from the oxidation of graphene, which is the crystalline allotrope of carbon [21]. On the other hand, activated carbons do not have well-defined crystallinity as these materials are typically produced by the thermal treatment (carbonization) of carbonaceous precursors, including biomass, coal and polymers [6]. Subsequently, the polymer matrices used in this work comprised commercial polyimide (Matrimid^®^ 5218) and in-house-produced polyimide (ODPA-TMPDA, where ODPA—4,4′-oxydiphthalic anhydride, and TMPDA—2,4,6-trimethyl-*m*-phenylenediamine). These polymers were selected due to their reasonably high CO_2_ permeabilities after being carbonized [15,22]. Meanwhile, in comparison to high free-volume polymers such as 6FDA-based (6FDA—4,4′-(hexafluoroisopropylidene)diphthalic anhydride) polyimides and PIM-1, Matrimid^®^ 5218 and ODPA-TMPDA polymers are relatively inexpensive and thus can be produced in a scalable manner. It should be noted that high molecular weight ODPA-TMPDA can be readily synthesized without rigorous monomer purifications [20]. The performance of the synthesized mixed-matrix CMSMs was evaluated by gas permeation tests with a CO_2_/N_2_ mixture as the feed gas to the membrane. The performances were benchmarked against the results reported in the literatures and the Robeson upper bound to quantify the enhancement in the gas separation performance.

## 2. Materials and Methods

### 2.1. Materials

Activated carbon (YP-50F) was purchased from Kuraray Chemical Co. (Tokyo, Japan). Matrimid^®^ 5218 polymer was purchased from Huntsman Corporation (Conroe, TX, USA). TMPDA, ODPA, acetic anhydride (Ac_2_O), graphene oxide (GO, 15–20 sheets, 4–10% edge-oxidized) and triethylamine (TEA) were purchased from Sigma Aldrich (St. Louis, MO, USA). Chloroform and *N,N*-dimethylacetamide (DMAc) were purchased from VWR. Distilled water was synthesized in-house. All chemicals and reagents mentioned above were used as received without additional purifications.

### 2.2. Synthesis of ODPA-TMPDA Polymer

**Figure 1:**
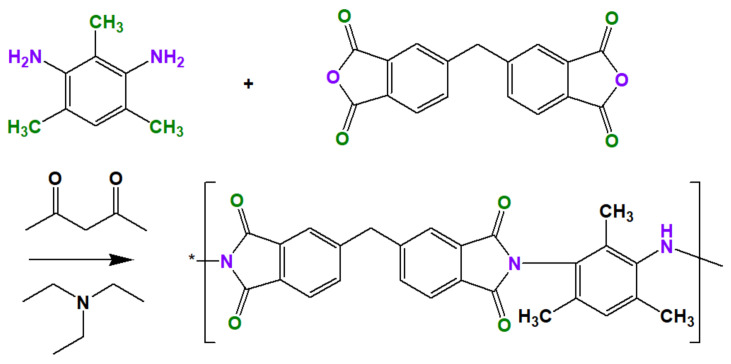
Reaction scheme for the synthesis of ODPA-TMPDA polymer.

The synthesis of ODPA-TMPDA polymer can be summarized by the reaction scheme in Figure 1 [23,24,25]. First, 20.0 g of DMAc was poured into a round-bottom flask, following which 1.63 g of TMPDA was added. During this process, sufficient agitation time was allocated to assure complete dissolution of TMPDA. This was followed by the addition of 3.36 g of ODPA to the solution. Vigorous stirring for at least 24 h was performed to create polyamic acid with 20 wt % concentration. Subsequently, imidization process was performed via the addition of 4.44 g of Ac_2_O and 4.39 g of TEA. Agitation for at least 24 h was conducted, followed by reprecipitation in ethanol solution. The unreacted substituents potentially present in the polymer were removed by washing with copious amount of ethanol. Finally, the polymer was dried in a vacuum oven at 120 °C. The structural properties of the as-synthesized ODPA-TMPDA polymer were characterized by Fourier Transform-Infrared Spectroscopy (FT-IR, Shimadzu, Kyoto, Japan) under ambient condition. The measurement was conducted in the wavenumber that ranged from 450–4000 cm^−1^ at the resolution of 4 cm^−1^.

### 2.3. Synthesis of Mixed-Matrix Carbon Molecular Sieve Membrane (Mixed-Matrix CMSM)

**Figure 2:**
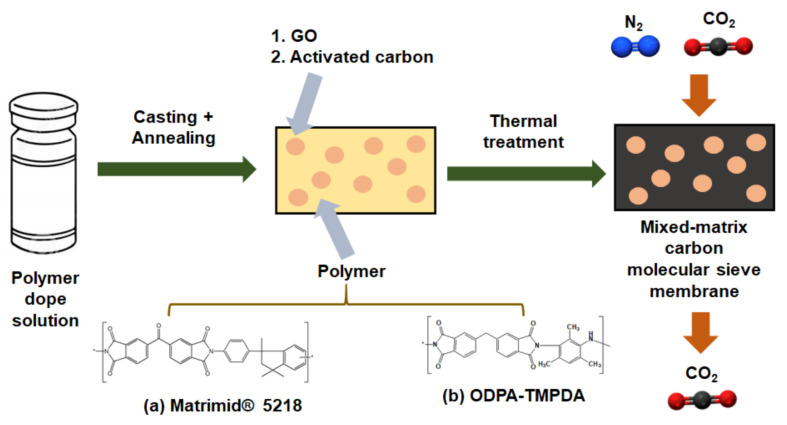
Synthesis scheme for the development of mixed-matrix carbon molecular sieve membranes containing (**1**) graphene oxide (GO) and (**2**) activated carbon derived from: (**a**) Matrimid^®^ 5218 and (**b**) ODPA-TMPDA polymers.

As illustrated in Figure 2, CMSMs were synthesized via the following procedure [22,26]. First, the polymer precursor membrane was prepared in a flat sheet configuration by solution-casting approach. A polymer dope solution was prepared by dissolving Matrimd^®^ 5218 and ODPA-TMPDA powders in chloroform. Then, a mixed-matrix polymer precursor membrane was fabricated by dispersing GO and YP-50F into the solution, using a sonication horn (Q125, Qsonica, Newton, CT, USA) to minimize the potential for particle aggregation. After sonication, the polymer was added to form the dope solution, which was then left agitated for a sufficient period before casting the membrane on a glass plate. The stability of the casting solution was investigated by taking photographic images at 12 h and 24 h after stopping agitation (Figure S1). The casting step was performed in a glovebox filled with chloroform vapor to prevent the rapid evaporation of chloroform from the dope solution. The membrane precursors were subsequently annealed in a vacuum oven at 160 °C overnight to remove any residual solvents present in the membrane. Next, the membrane precursors were carbonized to form CMSMs using a horizontal tube furnace (CTF 12/100/900, Carbolate GERO, Sheffield, United Kingdom). Before commencing the carbonization process, argon gas (99.9995% purity, Airliquide Singapore, Singapore) was purged throughout the quartz tube to allow the residual air or moisture potentially present in the tube to be removed effectively, for a duration of 1 h. This was followed by a two-step ramp-dwell thermal treatment, comprising heating at a 2 °C/min ramp rate from room temperature to 380 °C (dwelling for 0.5 h), followed by heating at a 0.5 °C/min ramp rate to 550 °C (dwelling for 2 h). After the thermal treatment, the carbonized membranes were allowed to cool to room temperature via natural convection. The resulting products were the CMSM samples.

### 2.4. Characterizations

The porosities of the GO and YP-50F were determined using N_2_ physisorption analysis at 77 K, conducted with the aid of a volumetric gas sorption analyzer (NOVATouch LX2, Quantachrome, Boynton Beach, FL, USA). Prior to the measurement, the samples were outgassed at 250 °C for 24 h under high vacuum. Subsequently, the pure component CO_2_ and N_2_ adsorption isotherms of GO and YP-50F were measured at 35 °C (measurement range of 0–1 bar), during which a water circulator was used to ensure an isothermal environment. Then, the samples were again outgassed at 250 °C for 24 h under high vacuum. The resulting gas adsorption isotherms were fitted using the single-site Langmuir equation (Equation (1)), where *q*, *q_sat_*, *b* and *p* are the adsorption quantity (mmol/g), saturation loading (mmol/g), Langmuir constant (bar^−1^) and pressure (bar), respectively. The fitting parameters are summarized in Tables S1 and S2. The CO_2_/N_2_ selectivity was calculated using Henry’s constant (*k_H_* = *q_sat_b*) and ideal adsorbed solution theory (IAST) as expressed in Equation (2), where *x*_1_ and *x*_2_ are the mole fractions of the adsorbed phase, whereas *y*_1_ and *y*_2_ are the mole fractions of the gas phase. The micropore size distributions of YP-50F, GO, carbonized Matrimid^®^, carbonized ODPA-TMPDA and mixed-matrix CMSMs were characterized by the measurement of CO_2_ uptake at 0 °C, with the use of density functional theory. The calculations were performed due to the difficulty in conducting N_2_ physisorption at 77 K for carbonized Matrimid^®^ 5218 and carbonized ODPA-TMPDA.

(1) $$q=\frac{q_{sat}\cdot b_{1}p}{1+b_{1}p}$$

(2) $$Selectivity=\;\frac{x_{1}/x_{2}}{y_{1}/y_{2}}$$

Using the Clausius–Clapeyron equation (Equation (3)), the isosteric heat of adsorption, *−Q_st_* for CO_2_ and N_2_, was calculated, where *p* is the pressure (bar), *T* is the absolute temperature (Kelvin) and *q* is the amount adsorbed (mmol/g). An explicit analytical solution for the calculation of *−Q_st_* using the single-site Langmuir equation has been reported in the literature [27,28,29]. Note that *−Q_st_* has been reported to be a weak function of temperature.

(3) $$-Q_{st}=RT^{2}\cdot {\left(\frac{\partial \ln p}{\partial T}\right)}_{q}$$

Next, the crystallinity of GO and YP-50F was determined using powdered X-ray diffraction (XRD). The diffractor used in this work was equipped with CuKα (1.5418 Å) radiation (Bruker, D2 Phaser, MA, USA). Under ambient condition, the diffractograms were measured in the 2*θ* range of 5°–40°, with a step size of 0.02°. The morphologies of GO, YP-50D and the mixed-matrix CMSMs were examined using field-emission scanning electron microscopy (FESEM; Joel, JSM6701, Tokyo, Japan) under an acceleration voltage of 5 kV.

### 2.5. Gas Permeation Analysis

A gas permeation system (GTR Tec Corporation) was utilized for the gas permeation tests under constant pressure-variable volume condition. The CO_2_/N_2_ mixture (test gas, CO_2_: 99.8% purity; N_2_: 99.995% purity) and helium gas (He: 99.995% purity) were purchased from Airliquide Singapore. First, the membrane was mounted onto the permeation cell in an isotherm environment at 35 °C. Throughout this process, the test gas and helium gas were supplied continuously at upstream and downstream, respectively, using a mass flow controller to control the flow rate. At a set time interval, the permeated gas was swept by helium, following which the measurement continued until no substantial fluctuation in the concentration of the permeated gas was observed. Then, the concentration of the permeated gas was calculated using gas chromatography. To verify the reproducibility of the gas permeation results, the measurements were conducted for at least three different samples of each CMSM and each mixed-matrix CMSM.

## 3. Results

### 3.1. GO and Porous Carbon Fillers

**Figure 3:**
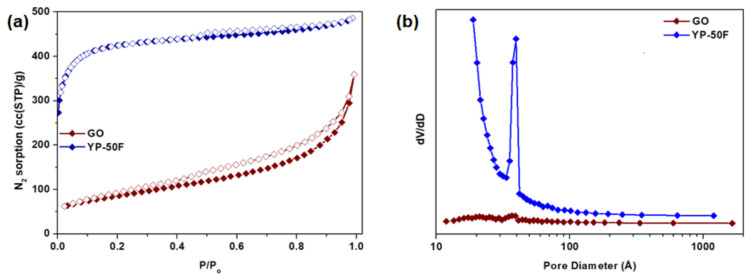
(**a**) N_2_ physisorption isotherms and (**b**) pore size distribution of GO and YP-50F at 77 K.

**Figure 4:**
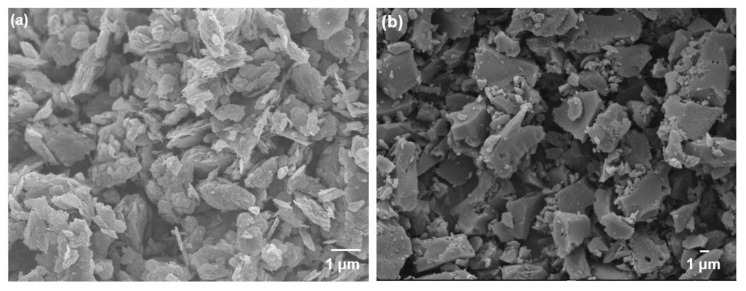
Field-emission scanning electron microscopy (FESEM) images of (**a**) GO and (**b**) YP-50F.

The surface areas and pore volumes of GO and YP-50F calculated from the N_2_ physisorption isotherms measured at 77 K (Figure 3) are summarized in Table 1. For YP-50F, a characteristic Type I isotherm [30] can be observed, indicating a high rate of N_2_ sorption at low P/P_o_, possibly due to enhanced adsorbent–adsorbate interaction in the narrow micropores. In contrast, GO has a Type III isotherm [30] that shows a lower N_2_ sorption rate, which can be attributed to its low accessible surface area and micropore volume. The presence of hysteresis between the adsorption and desorption isotherms of both GO and YP-50F indicates mesoporosity, based on the Barrett–Joyner–Halenda analysis, as demonstrated in Figure 3b. The structures of GO and YP-50F were further characterized by XRD, as shown in Figure S2. The signature peak of GO at c.a. 27° indicates its low oxidation level (4–10% edge-oxidized). As a heuristic based on previous studies, the characteristic peak of GO shifts to a lower angle with the increase in the degree of oxidation [1,3,21,31]. In contrast, the XRD pattern of YP-50F indicates its amorphous nature. Subsequently, the morphologies of GO and YP-50F were observed using FESEM as shown in Figure 4. Based on the images, it can be concluded that the GO consists of several layers (GO stacks) in platelet form, with the platelet size estimated to be around 1–3 μm. Meanwhile, YP-50F is highly irregular with particle sizes ranging from 3 to 6 μm.

### 3.2. CO_2_ and N_2_ Adsorption Properties of GO and YP-50F

**Figure 5:**
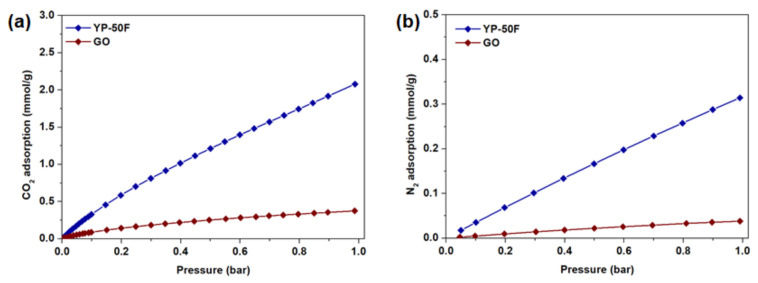
(**a**) CO_2_ and (**b**) N_2_ adsorption isotherms of GO and YP-50F at 35 °C.

The CO_2_ and N_2_ adsorption isotherms of GO and YP-50F measured at 35 °C under the pressure range of 0–1 bar are plotted in Figure 5. The isosteric heat of adsorption (−*Q_st_*) values of CO_2_ and N_2_ that were calculated based on the measurements at two different temperatures (25 and 35 °C) are summarized in Figure S3a, while the isotherms measured at 25 °C are included as supplementary information in Figure S3. Comparison of the CO_2_ and N_2_ adsorption isotherms reveals that both of the adsorbents demonstrate preferential adsorption of CO_2_ over N_2_, presumably due to its higher polarizability (CO_2_: 29.1 × 10^−25^ cm^3^; N_2_: 17.4 × 10^−25^ cm^3^) [32] and quadrupole moment (CO_2_: 4.3 × 10^−26^ esu cm^2^; N_2_: 1.5 × 10^−26^ esu cm^2^) [32]. The isotherms also indicate that YP-50F possesses higher CO_2_ and N_2_ adsorption capacities throughout the tested pressure range, which can be attributed to YP-50F possessing a higher surface area and micropore volume than GO. However, the CO_2_ adsorption profiles of both adsorbents are linear, which indicates the absence of strong binding sites, as depicted by the −*Q*_st_ calculation (Figure S4a). The CO_2_/N_2_ selectivity values calculated from IAST are relatively modest, being 21 and 9 for GO and YP-50F (Figure S4b), respectively (with a 1 bar CO_2_/N_2_ feed mixture of 20/80), in comparison to other reported porous materials such as zeolites and metal–organic frameworks (MOFs).

### 3.3. Fabrication of Mixed-Matrix CMSM with the Incorporation of GO and YP-50F

To confirm the successful synthesis of polyimide, the functional groups of ODPA-TMPDA were characterized by FT-IR analysis (Figure S5). From the FT-IR spectrum, the presence of C=O stretching in both symmetric and asymmetric form can be identified from the peaks at 1700 cm^−^^1^ and 1800 cm^−1^, respectively. The peak at 1300 cm^−1^ indicates C-N stretching. However, no peak at 3500 cm^−1^, which would correspond to the presence of residual polyamic acid, was observed in this polymer. The synthesis of ODPA-TMPDA involves a two-step procedure. Polyamic acid is first formed by the addition of its two monomers, which is subsequently followed by imidization. Thus, this FT-IR analysis confirmed that the potentially unreacted polyamic acid that could have been present in the polymer had been removed successfully after washing with copious ethanol. Note that our results are consistent to the observation in many previous studies [10,23,33].

**Figure 6:**
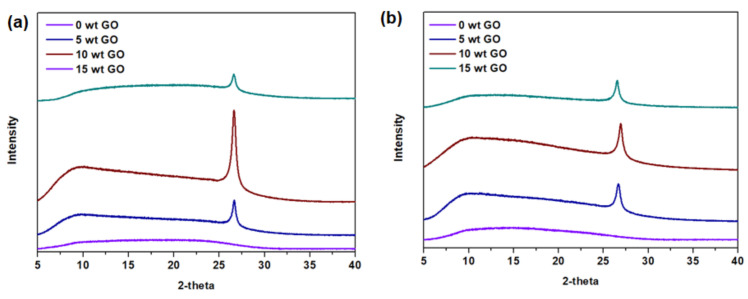
X-ray diffraction patterns of mixed-matrix CMSMs derived from (**a**) Matrimid^®^ 5218 and (**b**) ODPA-TMPDA membranes with systematic increase in GO loading.

The synthesis of mixed-matrix CMSM was conducted at a pyrolysis temperature of 550 °C. Thus, it was necessary to verify the thermal stability of the studied porous materials. As an initial step, thermogravimetric analysis (TGA) was conducted across the temperature range of 40–800 °C under pure nitrogen purging (Figure S6). Based on the TGA profiles, GO and YP-50F are thermally stable up to approximately 550 °C and 600 °C, which was sufficient for the development of the mixed-matrix CMSMs in this work. It should be noted that several well-reported porous materials, such as MOFs, generally suffer from poor thermal stability (loss of crystallinity at high temperature), making them infeasible for use in this study [6,12]. The initial decrease in the mass of GO and YP-50F is possibly attributable to the removal of residual water or solvents. The presence of adsorbents remaining after the carbonization process was investigated by the XRD measurement of GO (a crystalline material). In the profile (Figure 6), the characteristic peak of GO at 2*θ* = 27° can be detected, indicating that the crystallinity was not lost during the carbonization process (by comparing with the XRD pattern in Figure S2). Therefore, systematic increase in the loading of GO and YP-50F (5 wt %, 10 wt % and 15 wt %) was conducted in both the carbonized Matrimid^®^ 5218 and ODPA-TMPDA membranes.

**Figure 7:**
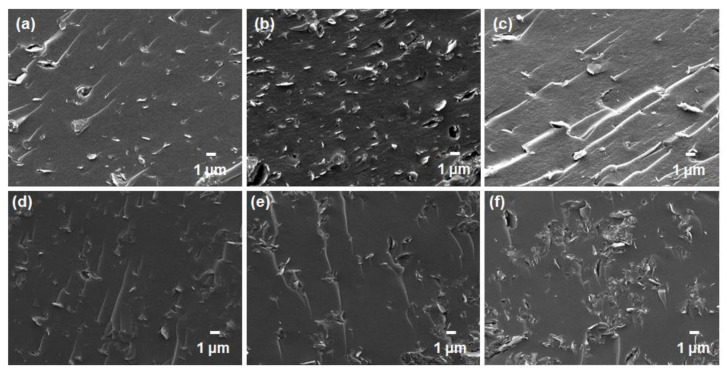

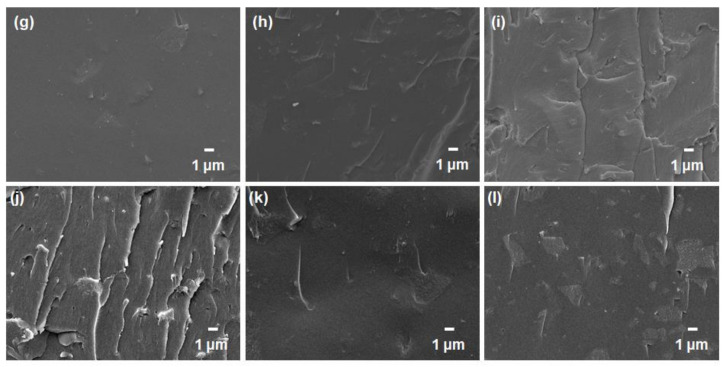
FESEM images of the mixed-matrix carbon molecular sieve membranes containing: (**a**) 5 wt %; (**b**) 10 wt % and (**c**) 15 wt % GO in Matrimid^®^ 5218; (**d**) 5 wt %; (**e**) 10 wt % and (**f**) 15 wt % GO in ODPA-TMPDA; (**g**) 5 wt %; (**h**) 10 wt % and (**i**) 15 wt % YP-50F in Matrimid^®^ 5218; (**j**) 5 wt %; (**k**) 10 wt % and (**l**) 15 wt % YP-50F in ODPA-TMPDA.

Subsequently, the cross-sectional morphologies of the mixed-matrix CMSMs with the addition of GO and YP-50F were studied by FESEM (Figure 7). It has been reported that inorganic-based adsorbents such as zeolites incorporated into typical mixed-matrix configurations generally do not achieve good compatibility or interfacial adhesion due to the pervasive “sieve-in-a-cage” phenomenon [34,35,36,37], which was further verified by taking SEM images at a higher magnification (Figure S7). In this study, however, the carbon-based materials (GO and YP-50F) did not evidently suffer such poor compatibility, as no visible voids were observed in the FESEM images. This may be attributable to the compatible functionalities between the polymer matrices and the carbon-based materials and to the thermal rearrangement of the polymer matrices during the carbonization process, which possibly healed any interfacial defects in the membrane [15,38].

### 3.4. Gas Permeation Properties

**Figure 8:**
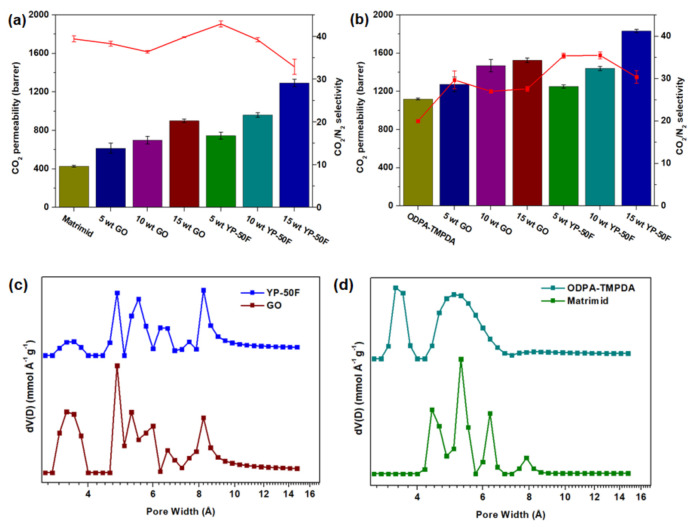
Gas permeation properties of GO- and YP-50F-based mixed-matrix CMSMs for CO_2_/N_2_ separation. The bar graphs indicate CO_2_ permeability, whereas the line graphs indicate CO_2_/N_2_ selectivity. (**a**) shows the results for membranes with Matrimid^®^ 5218 as the polymer precursor, whereas (**b**) shows the results for membranes with ODPA-TMPDA as the polymer precursor; (**c**) micropore size distribution of YP-50F and GO; (**d**) micropore size distribution of CMSMs (ODPA-TMPDA and Matrimid^®^).

The gas permeation properties of the mixed-matrix CMSMs were measured under 1 bar feed pressure (CO_2_/N_2_: 20/80) at 35 °C, and the results are summarized in Figure 8a,b. Based on the gas permeation data, the CMSMs derived from Matrimid^®^ 5218 and ODPA-TMPDA demonstrated an increase in CO_2_ permeability relative to their precursors reported in the literature [33]. Such enhancement has been reported in many previous studies, where carbonization increases the fractional free volume of membranes due to thermal rearrangement [15,26,38]. The gas separation performances of the mixed-matrix CMSMs were measured with incremental increases in the loading of GO and YP-50F in the respective membranes. Based on the gas permeation data, all the adsorbents showed substantially greater CO_2_ permeability than the pure CMSM, with improvement of 110%, 36%, 202% and 64% for 15 wt % GO and 15 wt % YP-50F in carbonized Matrimid^®^ 5218 and carbonized ODPA-TMPDA membranes, respectively. Moreover, none of the mixed-matrix CMSMs suffered from a major loss of CO_2_/N_2_ selectivity. The selectivity changes of the CMSMs containing 15 wt % GO and 15 wt % YP-50F in carbonized Matrimid^®^ 5218 and carbonized ODPA-TMPDA, relative to the pure CMSM, were 1%, −16%, 38% and 52%, respectively. Such enhancement can be possibly explained with the aid of micropore size distribution as reported in Figure 8c,d (in which the profiles were calculated from CO_2_ adsorption at 0 °C as reported in Figure S8). In general, the presence of additional micropores that are smaller than the pores in pure CMSM potentially contributes to an improved CO_2_/N_2_ selectivity [39,40]. Indeed, the presence of the smaller micropores was observed in the pore size distribution of mixed-matrix CMSMs **(**Figure S9). However, as larger pores (> 8 Å) were also observed in both fillers (YP-50F and GO) and mixed-matrix CMSMs, the capability of YP-50F and GO in improving CO_2_ permeability can be foreseen. Hence, based on the gas permeation results, it can be expected that the incorporation of carbon-based fillers of high porosity is a feasible method to assist the rapid transport of gas molecules through gas separation membranes, leading to an enhancement in gas permeability.

**Figure 9:**
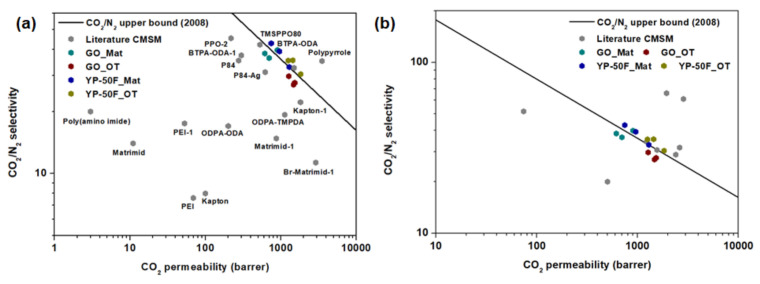
Robeson plot illustrating the performance of the proposed mixed-matrix CMSMs with reference to the literature data for **(a)** CMSMs [41,42,43,44,45,46,47,48,49,50] and **(b)** mixed-matrix CMSMs [51,52,53] (summarized in Table S3 and S4, respectively).

The performances of the mixed-matrix CMSMs were further benchmarked against the Robeson upper bound for CO_2_/N_2_ separation [13] and the available literature data for CMSMs and mixed-matrix CMSMs. The data are summarized in Figure 9 and Figure S10, respectively, with the tabulated data given in Tables S3 and S4. Based on the plots, the performance of the mixed-matrix CMSMs in this work surpassed the upper bound. The performance of the CMSMs in this work was far better than that of most CMSMs and mixed-matrix CMSMs reported to date. Thus, the composition of these CMSMs can be considered highly conducive to improving the gas separation performance of polymer membranes.

## 4. Conclusions

In this work, mixed-matrix CMSMs that contained YP-50F and GO in Matrimid^®^ 5218 and ODPA-TMPDA membranes were fabricated for investigation of their potential use in CO_2_/N_2_ separation. Based on the gas permeation data, both YP-50F and GO enhanced the CO_2_ permeability of the membranes substantially, as compared with the pure CMSM. In particular, for carbonized ODPA-TMPDA, enhancement in both CO_2_ permeability and CO_2_/N_2_ selectivity were achieved. This is attributed to the smaller micropore size in YP-50F and GO, which allowed enhancement in CO_2_/N_2_ selectivity, as well as the large pore volume, which allowed an improvement in CO_2_ permeability. Moreover, the performance of our mixed-matrix CMSMs surpassed the 2008 Robeson upper bound for CO_2_/N_2_ separation, indicating that membranes of this composition demonstrate strong improvement in gas separation performance.

## Figures and Tables

**Table 1:** Surface areas and pore volumes of (graphene oxide) GO and (activated carbon) YP-50F determined by N_2_ physisorption at 77K.

Sample	S_BET_^1^ (m^2^/g)	S_LANG_^1^ (m^2^/g)	S_micro_^2^ (m^2^/g)	V_micro_^2^ (cc^2^/g)	V_total_^3^ (cc/g)
GO	303	414	87	0.044	0.555
YP-50F	1462	1925	1377	0.632	0.987

^1^ Surface area (BET and Langmuir) were evaluated in the pressure range, P/P_o_ = 0.05–0.2. ^2^ Micropore properties (volume and surface area) were evaluated in the pressure range, P/P_o_ = 0.4–0.6 using the *t*-plot method. ^3^ The total pore volume was determined in the pressure P/P_o_ = 0.99.

## Data Availability

Not applicable.

## Supplementary Materials

The following are available online at https://www.mdpi.com/article/10.3390/membranes11040284/s1, Figure S1: Photographic images upon the incorporation of GO (left) and YP-50F (right) onto Matrimid^®^ 5218 (left) and ODPA-TMPDA (right) dope solution. The durations at which the photos were taken are: (**a**) 12 h and (**b**) 24 h, respectively, Figure S2: XRD of GO and YP-50F, Figure S3: (**a**,**b**) CO_2_ and N_2_ adsorption of GO and activated carbon (YP-50F) at 25 °C, Figure S4: (**a**) -*Q*_st_ of CO_2_ and N_2_ and (**b**) CO_2_/N_2_ IAST selectivity for GO and YP-50F (ratio of CO_2_/N_2_ in the feed is 20/80), Figure S5: FT−IR of ODPA-TMPDA polymer, Figure S6: TGA analysis of GO and activated carbon (YP-50F), Figure S7: FESEM images of mixed-matrix carbon molecular sieve membranes at higher magnification, that contains: (**a**) 5 wt %; (**b**) 10 wt % and (**c**) 15 wt % GO in Matrimid^®^ 5218; (**d**) 5 wt %; (**e**) 10 wt % and (**f**) 15 wt % GO in ODPA-TMPDA; (**g**) 5 wt %; (**h**) 10 wt % and (**i**) 15 wt % YP-50F in Matrimid^®^ 5218; (**j**) 5 wt %; (**k**) 10 wt % and (**l**) 15 wt % YP-50F in ODPA-TMPDA, Figure S8: CO_2_ uptake at 0 °C for YP-50F, GO, carbonized Matrimid^®^ 5218 and carbonized ODPA-TMPDA, Figure S9: Micropore size distribution of CMSMs and mixed-matrix CMSMs with: (**a**) Matrimid^®^ 5218 and (**b**) ODPA-TMPDA as the polymer precursor. The arrowhead in the figure dictates the presence of smaller micropore size in the mixed-matrix CMSM, Figure S10: Robeson plot that demonstrates CO_2_ permeabilities and CO_2_/N_2_ selectivity of CMSMs that are based on: (**a**) Matrimid^®^ 5218 and (**b**) ODPA-TMPDA polymeric precursors, Table S1: Fitting parameters for CO_2_ and N_2_ for GO and YP-50F at 25 °C, Table S2: Fitting parameters for CO_2_ and N_2_ for GO and YP-50F at 35 °C, Table S3: Performance of pure CMSM membranes that have been reported in the literature for CO_2_/N_2_ separation, Table S4: Performance of mixed-matrix CMSM membranes for CO_2_/N_2_ separation.
