# Supplementary material for: Carbon Molecular Sieve Membranes Comprising Graphene Oxides and Porous Carbon for CO2/N2 Separation
Source: Membranes (Basel). 2021 Apr 12;11(4):284. doi: 10.3390/membranes11040284 (PMC8069981; doi:10.3390/membranes11040284)
Supplement: Supplementary file 1 [file membranes-11-00284-s001.pdf]

membranes

Supplementary Materials: Effect of Incorporation of Carbon-Based Particles in Enhancing CO_2/N_2 Separation Performance in Carbon Molecular Sieve Membrane

Chong Yang Chuah [1], Junghyun Lee [2], Juha Song [2] and Tae-Hyun Bae [3],*

[1] Singapore Membrane Technology Centre, Nanyang Environment and Water Research Institute, Nanyang Technological University, Singapore 637141 Singapore; chongyang.chuah@ntu.edu.sg (C.Y.C.)
[2] School of Chemical and Biomedical Engineering, Nanyang Technological University, Singapore 637459 Singapore; junghyun002@e.ntu.edu.sg (J. L.), songjuha@ntu.edu.sg (J. S.)
[3] Department of Chemical and Biomolecular Engineering, Korea Advanced Institute of Science and Technology, Daejeon 34141, Republic of Korea
* Correspondence: thbae@kaist.ac.kr

SUMMARY

Number of pages: 8

Number of Figures: 10

Number of Tables: 4

Citation: Chuah, C.Y.; Lee, J.; Song, J.; Bae, T.-H. Carbon Molecular Sieve Membranes Comprising Graphene Oxides and Porous Carbon for CO_2/N_2 Separation. *Membranes* **2021**, *11*, 284. https://doi.org/10.3390/membranes11040284

Academic Editor: Adolfo Iulianelli

Received: 24 March 2021
Accepted: 9 April 2021
Published: 12 April 2021

Publisher's Note: MDPI stays neutral with regard to jurisdictional claims in published maps and institutional affiliations.

Copyright: © 2021 by the authors. Licensee MDPI, Basel, Switzerland. This article is an open access article distributed under the terms and conditions of the Creative Commons Attribution (CC BY) license (http://creativecommons.org/licenses/by/4.0/).

Table S1. Fitting Parameters for CO_2 and N_2 adsorptions onto GO and YP-50F at 25 °C.

Sample	*Gas*	q_{sat}	*b*	R^2 value	Henry's Constant, k_H
GO	CO_2	0.6759	1.762	0.9942	1.191
	N_2	0.6759	0.07684	0.9943	0.05194
YP-50F	CO_2	6.087	0.7079	0.9991	4.309
	N_2	6.087	0.06686	0.9992	0.4070

Unit of P—bar; Unit of *q*—mmol/g

Table 2S. Fitting Parameters for CO_2 and N_2 adsorptions onto GO and YP-50F at 35 °C.

Sample	*Gas*	$q_{sat,1}$	*b*	R^2 value	Henry's Constant, k_H
GO	CO_2	0.5976	1.541	0.9959	0.9209
	N_2	0.5976	0.07229	0.9915	0.04320
YP-50F	CO_2	5.854	0.535	0.9987	3.132
	N_2	5.854	0.05791	0.9996	0.3390

Unit of P—bar; Unit of *q*—mmol/g

Table S3. Performance of pure CMSMs that reported in the literature for CO_2/N_2 separation.

Polymer Matrix	Carbonization Condition	Measurement Condition	CO_2 Permeability (Barrer)	CO_2/N_2 Selectivity	Ref.
**BTPA-ODA**	550 °C, Argon	25 °C, Pure gas	1500	32.6	[1]
**BTPA-ODA-1**	700 °C, Argon	25 °C, Pure gas	300	37.5	[1]
**Br-Matrimid-1**	550 °C, Vacuum	35 °C, 10 atm, Pure gas	2900	11.3	[2]
**Kapton**	550 °C, Vacuum	25 °C, Pure gas	100	8.0	[3]
**Matrimid**	475 °C, Vacuum	25 °C, Pure gas	11	14.0	[3]
**Matrimid-1**	550 °C, Vacuum	35 °C, 10 atm, Pure gas	871	14.8	[2]
**Matrimid-2**	550 °C, Argon	35 °C, mixed gas (20/80)	428	39.4	This work
**ODPA-TMPDA**	550 °C, Argon	35 °C, mixed gas (20/80)	1120	20.0	This work
**Kapton-1**	600 °C, Vacuum	35 °C, Pure gas	1820	22.2	[4]
**ODPA-ODA**	650 °C	50 °C, Pure gas	201	17.0	[5]
**P84**	600 °C, Nitrogen	Pure gas	276	35.4	[6]
**P84-Ag**	600 °C, Nitrogen	Pure gas	619	31.1	[6]
**PEI**	550 °C, Vacuum	25 °C, Pure gas	69	7.6	[7]
**PEI-1**	500 °C, Vacuum	26 °C, Pure gas	53	17.5	[8]
**Polypyrrole**	550 °C, Nitrogen	35 °C, Pure gas	3520	35.2	[9]
**Poly(amino imide)**	150 °C, Nitrogen	35 °C, Pure gas	3	20.0	[9]
**PPO-2**	650 °C, Vacuum	25 °C, Pure gas	218	45.4	[10]

Note: BTDA—Benzophenone tetracarboxylic dianhydride; ODA—4,4′-oxydianiline; PPO—poly(p-phenylene oxide); TMS—chlorotrimethylsilane.

Table S4. Performance of mixed-matrix CMSMs for CO_2/N_2 separation.

Membranes	Measurement Condition	CO_2 Permeability (Barrer)	CO_2/N_2 Selectivity	F_{index}	Ref.
15 wt% GO (Matrimid)	35 °C, mixed gas (21/79)	900	39.8	0.77	This work
15 wt% GO (ODPA-TMPDA)	35 °C, mixed gas (21/79)	1526	27.6	1.24	This work
15 wt% YP-50F (Matrimid)	35 °C, mixed gas (21/79)	1294	32.9	0.59	This work
15 wt% YP-50F (ODPA-TMPDA)	35 °C, mixed gas (21/79)	1833	30.4	1.70	This Work
30 wt% PS-MFI (ODPA-TMPDA)	35 °C, mixed gas (20/80)	2397	28.8	1.81	This work
30 wt% ETS-10 (ODPA-TMPDA)	35 °C, mixed gas (20/80)	1234	34.2	1.59	This work
30 wt% SAPO-34 (ODPA-TMPDA)	35 °C, mixed gas (20/80)	2615	31.7	2.15	This work
15 wt% FS (ODPA-TMPDA)	35 °C, mixed gas (20/80)	1560	30.7	1.54	This work
24 wt% SBA-15 (PAA)	25 °C, mixed gas (50/50)	1939	66	0.14	[11]
24 wt% MCM-48 (PAA)	25 °C, mixed gas (50/50)	2850	61	0.31	[11]
3 wt% Zeolite L (PFA)	25 °C, pure gas	503	20	2.97	[12]
2 wt% Zeolite T (PFA)	25 °C, pure gas	74	51.6	1.45	[13]

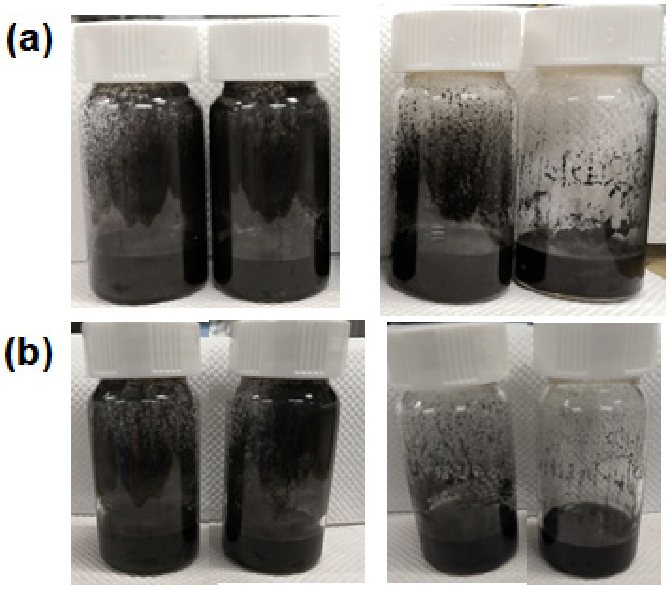

Figure S1. Photographic images upon the incorporation of GO (left) and YP-50F (right) onto Matrimid® 5218 (left) and ODPA-TMPDA (right) dope solution. The photos were taken at (**a**) 12 h and (**b**) 24 h after stopping agitation, respectively.

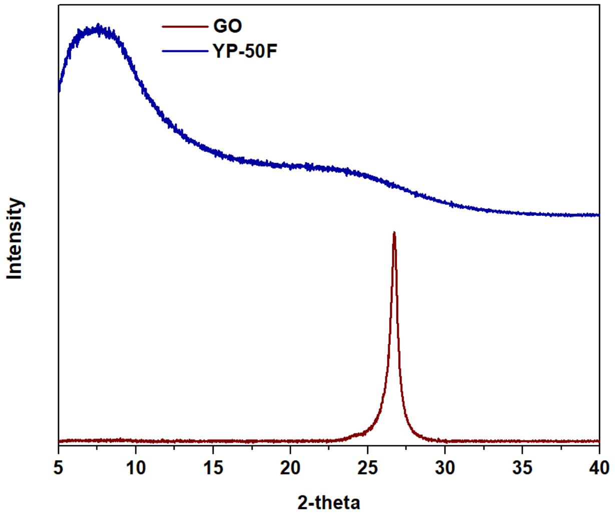

Figure S2. XRD of GO and YP-50F.

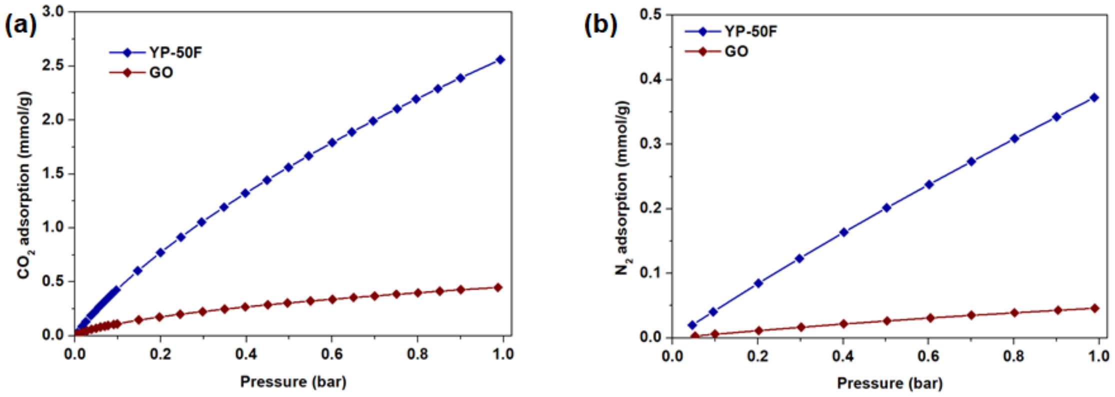

Figure S3. (**a**, **b**) CO_2 and N_2 adsorptions on GO and activated carbon (YP-50F) at 25 °C.

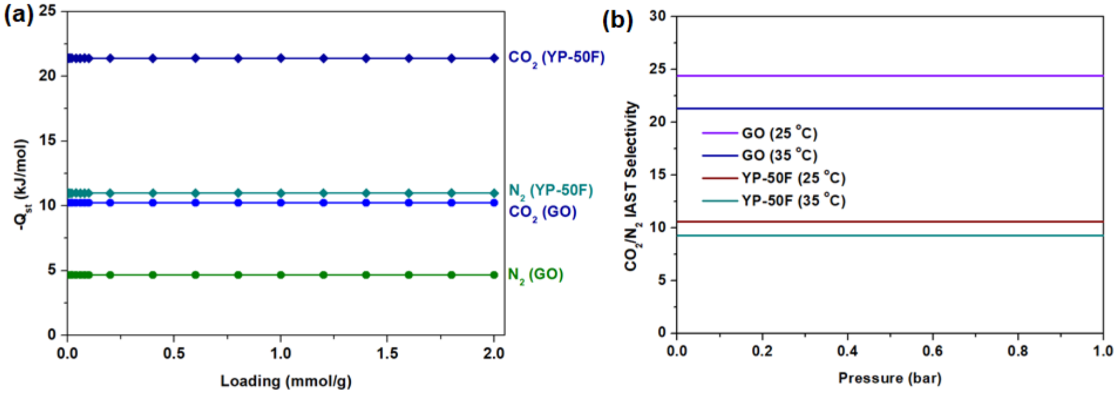

Figure S4. (a) The isosteric heats of adsorption ($-Q_{st}$) of CO_2 and N_2 and **(b)** CO_2/N_2 IAST selectivity for GO and YP-50F (the ratio of CO_2/N_2 in the feed is 20/80).

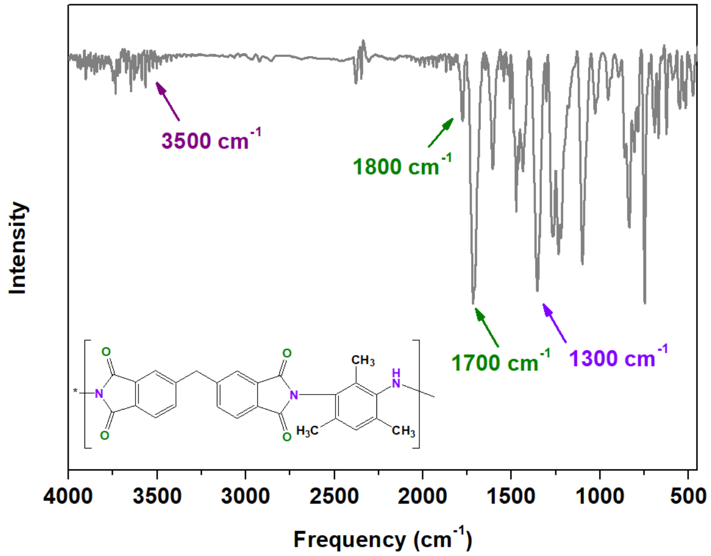

Figure S5. FT−IR spectrum of ODPA-TMPDA polymer.

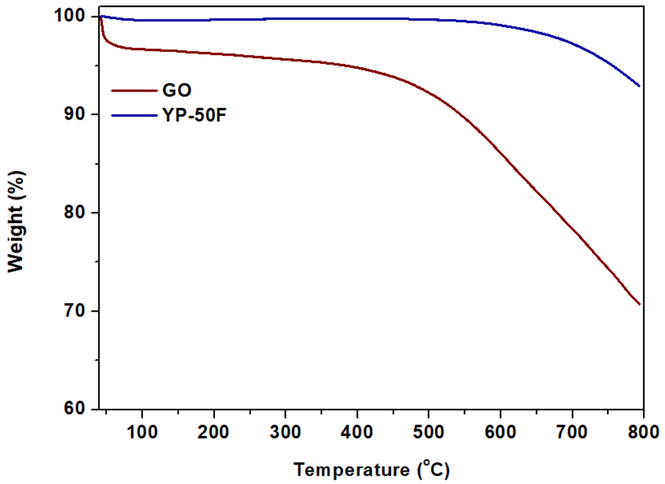

Figure S6. The TGA curves of GO and activated carbon (YP-50F).

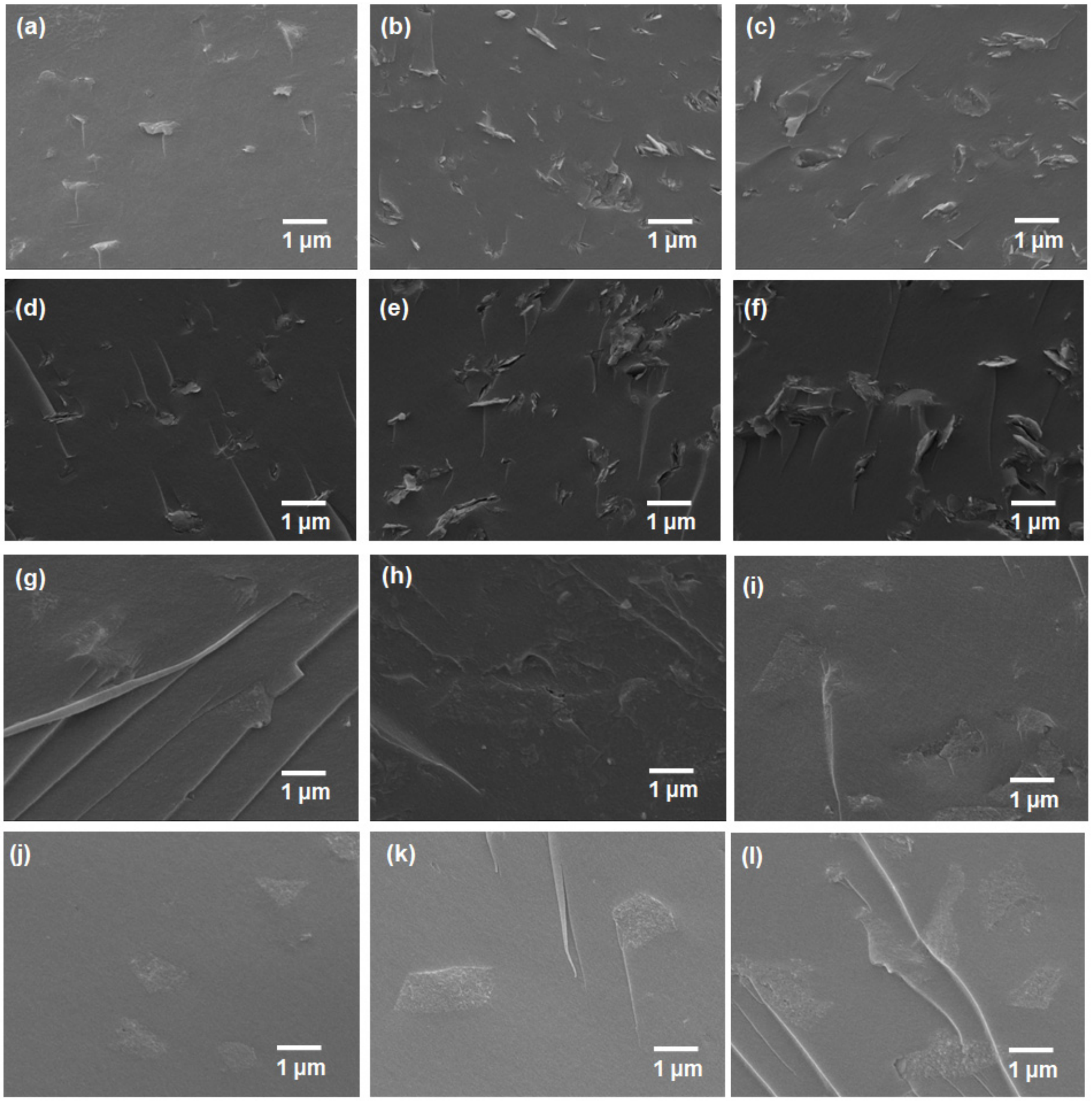

Figure S7. FESEM images of mixed-matrix carbon molecular sieve membranes at higher magnification. (**a, b, c**) 5 wt%, 10 wt% and 15 wt% GO in Matrimid® 5218; (**d, e, f**) 5 wt%, 10 wt% and 15 wt% GO in ODPA-TMPDA; (**g, h, i**b 5 wt%, 10 wt% and 15 wt% YP-50F in Matrimid® 5218; (**j, k, l**) 5 wt%, 10 wt% and 15 wt% YP-50F in ODPA-TMPDA.

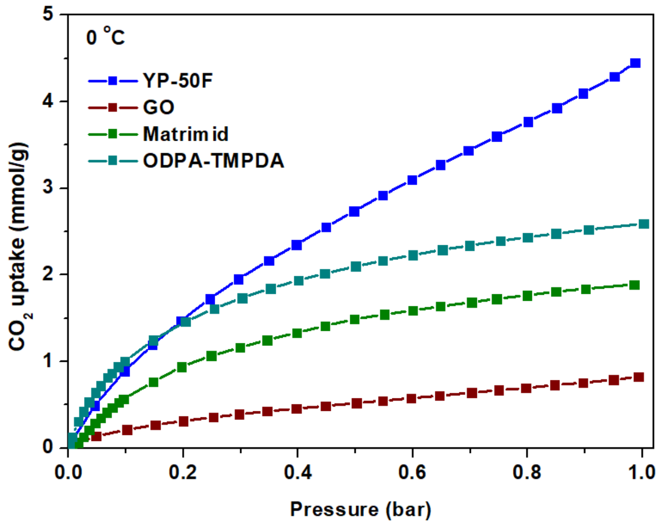

Figure S8. The CO_2 uptakes at 0 °C for YP-50F, GO, carbonized Matrimid® 5218 and carbonized ODPA-TMPDA.

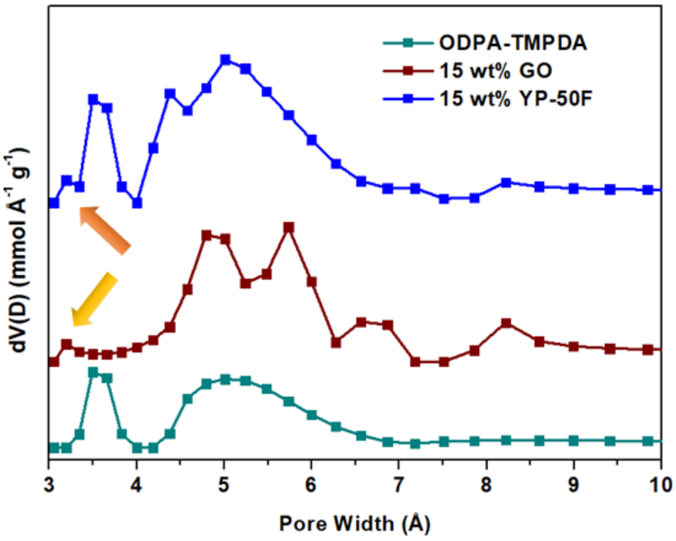

Figure S9. Pore size distribution of CMSMs and mixed-matrix CMSMs with ODPA-TMPDA as the polymer precursor. The arrowhead indicated in the figure shows the presence of smaller micropores in the mixed-matrix CMSMs.

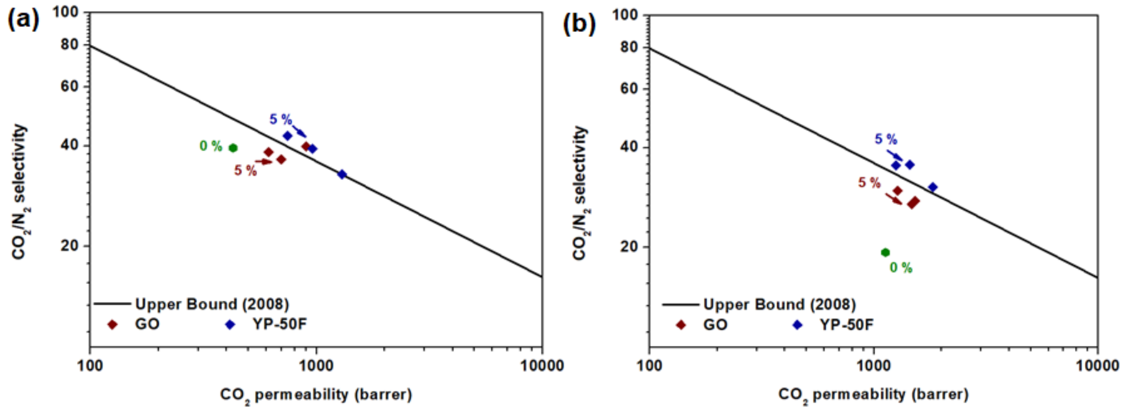

Figure S10. Robeson plot that demonstrates CO_2 permeabilities and CO_2/N_2 selectivity of CMSMs that are based on (**a**) Matrimid® 5218 and (**b**) ODPA-TMPDA polymeric precursors.

References

1. Kim, Y.K.; Park, H.B.; Lee, Y.M. Carbon molecular sieve membranes derived from thermally labile polymer containing blend polymers and their gas separation properties. *J. Membr. Sci.* **2004**, *243*, 9–17, doi:10.1016/j.memsci.2004.05.001.
2. Xiao, Y.; Dai, Y.; Chung, T.-S.; Guiver, M.D. Effects of Brominating Matrimid Polyimide on the Physical and Gas Transport Properties of Derived Carbon Membranes. *Macromol.* **2005**, *38*, 10042–10049, doi:10.1021/ma051354j.
3. Fuertes, A.; Nevskaia, D.; Centeno, T. Carbon composite membranes from Matrimid® and Kapton® polyimides for gas separation. *Microporous Mesoporous Mater.* **1999**, *33*, 115–125, doi:10.1016/s1387-1811(99)00129-8.
4. Suda, H.; Haraya, K. Gas Permeation through Micropores of Carbon Molecular Sieve Membranes Derived from Kapton Polyimide. *J. Phys. Chem. B* **1997**, *101*, 3988–3994, doi:10.1021/jp963997u.
5. Zhang, B.; Wu, Y.; Lu, Y.; Wang, T.; Jian, X.; Qiu, J. Preparation and characterization of carbon and carbon/zeolite membranes from ODPA–ODA type polyetherimide. *J. Membr. Sci.* **2015**, *474*, 114–121, doi:10.1016/j.memsci.2014.09.054.
6. Barsema, J.; Balster, J.; Jordan, V.; Van Der Vegt, N.; Wessling, M. Functionalized Carbon Molecular Sieve membranes containing Ag-nanoclusters. *J. Membr. Sci.* **2003**, *219*, 47–57, doi:10.1016/s0376-7388(03)00176-5.
7. Itta, A.K.; Tseng, H.-H.; Wey, M.-Y. Effect of dry/wet-phase inversion method on fabricating polyetherimide-derived CMS membrane for H2/N2 separation. *Int. J. Hydrogen Energy* **2010**, *35*, 1650–1658, doi:10.1016/j.ijhydene.2009.12.069.
8. Rao, P.S.; Wey, M.-Y.; Tseng, H.-H.; Kumar, I.A.; Weng, T.-H. A comparison of carbon/nanotube molecular sieve membranes with polymer blend carbon molecular sieve membranes for the gas permeation application. *Microporous Mesoporous Mater.* **2008**, *113*, 499–510, doi:10.1016/j.micromeso.2007.12.008.
9. Kita, H.; Yoshino, M.; Tanaka, K.; Okamoto, K.-I. Gas permselectivity of carbonized polypyrrolone membrane. *Chem. Commun.* **1997**, 1051–1052, doi:10.1039/a700048k.
10. Yoshimune, M.; Fujiwara, I.; Haraya, K. Carbon molecular sieve membranes derived from trimethylsilyl substituted poly(phenylene oxide) for gas separation. *Carbon* **2007**, *45*, 553–560, doi:10.1016/j.carbon.2006.10.017.
11. Li, L.; Wang, T.; Liu, Q.; Cao, Y.; Qiu, J. A high CO2 permselective mesoporous silica/carbon composite membrane for CO2 separation. *Carbon*, **2012**, *50*, 5186–5195.
12. Yin, X.; Wang, J.; Chu, N.; Yang, J.; Lu, J.; Zhang, Y.; Yin, D. Zeolite L/carbon nanocomposite membranes on the porous alumina tubes and their gas separation properties. *J. Membr. Sci.* **2010**, *348*, 181–189, doi:10.1016/j.memsci.2009.10.055.
13. Yin, X.; Chu, N.; Yang, J.; Wang, J.; Li, Z. Thin zeolite T/carbon composite membranes supported on the porous alumina tubes for CO2 separation. *Int. J. Greenh. Gas Control.* **2013**, *15*, 55–64, doi:10.1016/j.ijggc.2013.01.032.
